# Prognosis stratification in breast cancer and characterization of immunosuppressive microenvironment through a pyrimidine metabolism-related signature

**DOI:** 10.3389/fimmu.2022.1056680

**Published:** 2022-11-29

**Authors:** Yongzhou Luo, Wenwen Tian, Xiuqing Lu, Chao Zhang, Jindong Xie, Xinpei Deng, Yi Xie, Shuhui Yang, Wei Du, Rongfang He, Weidong Wei

**Affiliations:** ^1^ Department of Breast Oncology, Sun Yat-sen University Cancer Center, State Key Laboratory of Oncology in South China, Collaborative Innovation Center for Cancer Medicine, Guangzhou, China; ^2^ Surgical and Transplant Intensive Care Unit of The Third Affiliated Hospital, The Third Affiliated Hospital of Sun Yat-sen University, Guangzhou, China; ^3^ Department of Pathology, The First People’s Hospital of Changde City, Changde, Hunan, China; ^4^ Department of Pathology, The First Affiliated Hospital, Hengyang Medical School, University of South China, Hengyang, Hunan, China

**Keywords:** pyrimidine metabolism, breast cancer, prognosis signature, tumor immune microenvironment, therapy resistance, immunotherapy

## Abstract

Pyrimidine metabolism is a hallmark of cancer and will soon become an essential part of cancer therapy. In the tumor microenvironment, cells reprogram pyrimidine metabolism intrinsically and extracellularly, thereby promoting tumorigenesis. Metabolites in pyrimidine metabolism have a significant impact on promoting cancer advancement and modulating immune system responses. In preclinical studies and practical clinical applications, critical targets in pyrimidine metabolism are acted upon by drugs to exert promising therapeutic effects on tumors. However, the pyrimidine metabolism in breast cancer (BC) is still largely underexplored. In this study, 163 credible pyrimidine metabolism-related genes (PMGs) were retrieved, and their somatic mutations and expression levels were determined. In addition, by using The Cancer Genome Atlas (TCGA) and the Molecular Taxonomy of Breast Cancer International Consortium (METABRIC) databases, 12 PMGs related to the overall survival (OS) were determined using the univariate Cox regression analysis. Subsequently, by performing the LASSO Cox hazards regression analysis in the 12 PMGs in TCGA-BRCA dataset, we developed a prognosis nomogram using eight OS-related PMGs and then verified the same in the METABRIC, GSE96058, GSE20685, GSE42568 and GSE86166 data. Moreover, we validated relationships between the pyrimidine metabolism index (PMI) and the survival probability of patients, essential clinical parameters, including the TNM stage and the PAM50 subtypes. Next, we verified the predictive capability of the optimum model, including the signature, the PAM50 subtype, and age, using ROC analysis and calibration curve, and compared it with other single clinical factors for the predictive power of benefit using decision curve analysis. Finally, we investigated the potential effects of pyrimidine metabolism on immune checkpoints, tumor-infiltrating immune cells, and cytokine levels and determined the potential implications of pyrimidine metabolism in BC immunotherapy. In conclusion, our findings suggest that pyrimidine metabolism has underlying prognostic significance in BC and can facilitate a new management approach for patients with different prognoses and more precise immunotherapy.

## Introduction

Breast cancer (BC) is presently one of the most prevalent malignancies. Furthermore, it is the top cause of cancer-related fatalities among the female population (1). Though the treatment of BC has evolved significantly in recent years, and the treatment efficacy has also improved remarkably, some patients continue to have poor treatment outcomes due to their susceptibility to recurrence, metastasis, and drug resistance. One of the most notable issues leading to this result is the heterogeneity of BC (2). Thus, the medical field requires new bio-indicators and treatment targets to guide the therapeutic management of BC patients.

Over the past half-century, the oncogene revolution has led to a large volume of research and a series of mutational events causing key phenotypes in tumor cells to cleverly combine and alter multiple signaling pathways. In addition, high-throughput sequencing technology revealed the presence of more diverse mutations associated with tumorigenesis and progression than previously assumed (3, 4). These mutations impacted a host of critical signal pathways and processes that converge to accommodate tumor metabolism and support tumor growth and migration. Some of these metabolic alterations, which were vital for the malignant transformation of tumors, were closely associated with the malignant phenotype of tumors (5). Therefore, we hypothesize that these metabolic alterations can function as a crucial hallmark of survival prognosis in tumor patients.

During tumor cell proliferation, the need to synthesize ribosomal RNA (rRNA), replicate the genome (synthesize DNA), and maintain the transcriptome (produce large amounts of mRNA) increases the demand for nucleotides. As the exogenous intake of nucleotides is essentially negligible, the endogenous synthesis of nucleotides is more important than that of other nutrients. Pyrimidines, in turn, are an essential and important component of nucleotides, and therefore, pyrimidine metabolism has a significant impact on the advancement of neoplasms (6). Accumulating evidence suggests that pyrimidine metabolism is pivotal in the progression of several kinds of carcinomas and the development of drug resistance. For example, blocking pyrimidine synthesis enhanced the molecular therapeutic response of glioblastoma stem cells (7). Adaptive response of myeloid malignancy cells to pyrimidine metabolic network led to resistance to decitabine and 5-azacytidine (8). Dihydroorotate dehydrogenase (DHODH)-driven pyrimidine biosynthesis was one of the major mechanisms linking respiration and tumorigenesis. Not only that, DHODH inhibitors were also potential anticancer medicines (9). Reprogramming of CDA-mediated pyrimidine metabolism under ER stress provides a survival advantage for the dehydrogenase-driven hyperactivation of pyrimidine MUC1 oncoprotein (10). Furthermore, pyrimidine metabolism has a non-proliferative role *via* the epithelial-to-mesenchymal transition in several epithelial and non-epithelial tumors (11).

Tumor-infiltrating immune cells (TIIs), an essential component of the tumor microenvironment (TME), are a series of immune effector and immune suppressor cells in and around the neoplasm (12). In addition, metabolic disorders in the TME, especially the recently discovered changes in pyrimidine metabolism, have a critical impact on tumor development (13). Pyrimidine *de novo* synthesis has been the target of a range of chemotherapy drugs in widespread use (e.g., 5-fluorouracil) and immunosuppressant drugs (e.g., leflunomide and brequinar), emphasizing its importance in cancer progression and immune regulation (14). Leflunomide-treated tumors that inhibit a key enzyme for ab initio synthesis of pyrimidine exhibited reduced CTLA-4+ T cells, suggesting reduced intra-tumor T cell depletion and possibly increased anti-tumor immunity. However, the researchers did not investigate the effect of the combination with immune checkpoint inhibitors, for example, inhibitors targeting programmed cell death-1 (PD-1), programmed cell death ligand-1 (PD-L1), and cytotoxic T-lymphocyte antigen-4 (CTLA-4) (15). DHODH and mitochondria-associated pyrimidine synthesis were independent and important cytostatic regulators of activated T cells (16). Studies have demonstrated that a large number of tumor-associated macrophages (TAMs) infiltrating pancreatic ductal adenocarcinoma release a series of pyrimidines, including deoxycytidine, which competes for the inhibition of gemcitabine through multiple mechanisms (17). Nevertheless, systematic studies on the interactions between pyrimidine metabolism and TME are still insufficient.

In this study, we investigated the prognostic value of pyrimidine metabolism-related genes (PMGs) and established a signature that could be used to predict the survival prognosis and immunotherapy benefits of BC patients. We further conducted a comprehensive evaluation of the clinical application of this signature. Additionally, potential correlations between this feature and TME landforms were revealed. This comprehensive analysis might provide new insights on pyrimidine metabolism and immunotherapy for cancer research.

## Materials and methods

### Patients data collection

The transcriptome expression matrices and corresponding clinicopathological information of BC were retrieved from The Cancer Genome Atlas (TCGA) database (113 normal breast samples and 1,113 BC samples), the Molecular Taxonomy of Breast Cancer International Consortium (METABRIC) database (1,904 BC samples), and GSE96058 (3,409 BC samples), GSE20685 (327 BC samples), GSE42568 (104 BC samples), GSE86166 (366 BC samples) from the Gene Expression Omnibus (GEO) database. We obtained 920 patients of TCGA-BRCA as a training cohort and 1,891 patients of METABRIC cohort, 3,069 patients of GSE96058 cohort, 327 patients of GSE20685 cohort, 104 patients of GSE42568 with 366 patients of GSE86166 cohort as validation sets whose overall survival (OS) was more than 30 days. In total, 186 pyrimidine metabolism-relevant genes were obtained from the Molecular Signature Database v7.5.1 (MSigDB). In addition, after taking the intersection of the above 186 PMGs with the overall genes in the TCGA-BRCA, METABRIC and GSE96058 datasets mentioned above, 163 overlapping PMGs were extracted for further analysis **(** **Supplementary Figure 1** **)**.

### Identification of somatic mutations and variations in the expression of genes among PMGs

We downloaded somatic mutations of BC patients in the training cohort from the UCSC Xena database. The somatic mutation frequencies of 163 PMGs are shown in the waterfall plot evaluated using the R package “maftools” (18). Differentially expressed genes (DEGs), which are related to pyrimidine metabolism, were determined using the R package “edgR” (19).These significant DEGs are demonstrated in a volcano plot along with a heatmap.

### Acquisition of OS-related PMGs

PMGs significant for predicting survival prognosis were ascertained using the univariate Cox hazard regression analysis in TCGA-BRCA and METABRIC cohorts. The overlapping OS-related PMGs were extracted to construct the next prognostic model. Then, the mRNA expression levels and locations on the chromosomes of these genes were visualized using the R package “RCircos” (20), and the correlations between these genes and other genes were shown with strings. Besides, correlation matrix plots were constructed to ascertain the correlation features between these overlapping OS-related PMGs.

### Construction and validation of pyrimidine metabolism-related prognostic signature

For the further discovery of candidate PMGs with more prognostic significance, the least absolute shrinkage and selection operator (LASSO) Cox regression analysis was applied to the training cohort (21). Then, utilizing the “glmnet” R package (22), the eight optimized PMGs were explored to model the prognosis of BC patients. Their mRNA expression level between BC tissues and normal mammary tissues was plotted in a boxplot, and their prognostic significance for OS were described in a survival curve. In accordance with the prediction model, the pyrimidine metabolic index (PMI) can be derived for patients by applying the following formula.


PMI = ∑Ei*γi

(Ei represents the mRNA expression level of each PMG; and γi represents the corresponding regression coefficient)

Median-split was applied to classify BC patients in each cohort into high PMI group and low PMI group. The classification accuracy of the signatures was evaluated based on principal component analysis (PCA).

### Comprehensive evaluation of PMI and clinical parameters in BC patients

To further demystify the applicability of PMI to actual clinical problems, boxplots of Kruskal’s test were employed to compare the differences in PMI values across the various clinicopathological parameters in the TCGA-BRCA, METABRIC and GSE96058 datasets to varying degrees. In addition, the heatmap demonstrated the association between the mRNA expression levels of PMGs included in the signature and several clinical indicators, including PMI, T stage, N stage, American Joint Committee on Cancer (AJCC) stage, and survival status in the training set, as well as PMI, tumor size, positive lymph nodes, PAM50 subtype, AJCC stage, grade, and survival status in the two validation datasets.

### Construction and assessment of pyrimidine metabolism-relevant clinical nomogram

Next, univariate and multivariate Cox regression analyses were performed to characterize whether PMI was an independent prognostic factor for BC patients. On the basis of these results, we developed a clinicopathological nomogram related to pyrimidine metabolism by utilizing the R packages “rms” and “regplot” (23), which combined PMI with two additional clinical features, age and PAM50 subtypes, in the training set. Verification of the predictive power of the nomogram was completed by the analysis of calibration curves (24) and decision curve analysis (DCA) which were plotted. R package “timeROC” was used to performed the Receiver Operating Characteristic (ROC) analyses (25).

### Illustration of the differential biofunction and metabolic network within the two PMI groups

To illustrate the differences in biological functions and metabolic network between the high and low PMI groups, gene set enrichment analysis (GSEA) was performed (26). “c5.all.v7.5.1.symbols.gmt” [GO] and “c2.cp.kegg.v7.5.1.symbols.gmt” [KEGG] were selected as the reference molecular signature database, and |NES| > 1.5 and FDR q-values< 0.1 were considered to be statistically significant (27).

### Prospective implications for immunotherapy and tumor immune microenvironment landscape estimates based upon PMI

In the past half-century, with the advent of immune checkpoint inhibitors, great progress has been made in the clinical immunotherapy of BC. Therefore, the mRNA expression levels of immune checkpoints, including PD-1, PD-L1, CTLA4, CD28, CD226, IDO1, TIGIT, and PVR in the high- and low-PMI groups were compared with the Wilcox test to initially validate the potential significance of PMI-based immunotherapy.

The estimate scores, immune scores and stromal scores were evaluated using the ESTIMATE algorithm (28) to further analyze the TME landscape between the two PMI subgroups. Twenty-two tumor immune-infiltrating cell types in the TME in the TCGA-BRCA, METABRIC and GSE96058 cohorts of BC tissues were computed using the CIBERSORT deconvolution algorithm (29). To elucidate the association between PMI and cytokines in TME, we selected several key cytokines whose mRNA expression levels were compared in the high- and low-PMI groups, including IL-1B, IL-6, IL-10, IL-15, IL-27, IL-33, INFG, and TNF.

### Cell culture

Cell lines of human BC including BT-549, MCF-7, T47D, MDA-MB-231 and SK-BR-3 were obtained from ATCC (American Type Culture Collection). All cells were grown at 37°C in a 5% CO_2_ and 70% relative humidity atmosphere without antibiotics. Survival cells were passaged for less than six months before testing negative for mycoplasma (30).

### RNA isolation and quantitative real-time PCR analysis

This technique was applied using RNA Quick Purification Kit. An overview of primer sequences is provided in **Supplementary Table S1**. The samples were analyzed in triplicate using a Bio-Rad CFX96 system with SYBR Green. The qRT-PCR plate was supplied by NEST. An expression level of RNA was calculated with 2^−ΔΔCT^ and normalized to β-actin.

### Statistical analyses

R software (Version 4.2.0) was utilized to perform all statistical analyses. The differences between the two groups were examined by the Wilcox test, and Kruskal–Wallis test was used for cases that involved more than two groups. The Kaplan–Meier (KM) curve was achieved by log rank test. Screening of PMG and independent OS prognostic indicators associated with OS in BC was performed using univariate and multivariate Cox regression analyses. The correlation matrix was graphed based on Spearman’s correlation test. Bilateral and p< 0.05 were ascertained to have statistical significance.

## Results

### Identification of prognostic pyrimidine metabolism-relevant genes in BC patients

**Figure 1:**
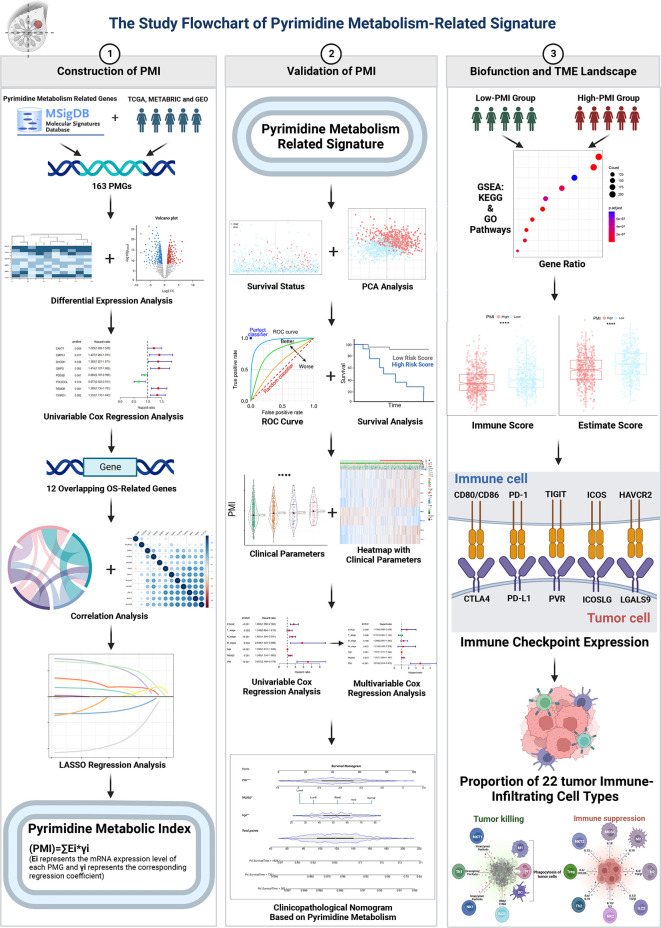
Study flowchart.

**Figure 2:**
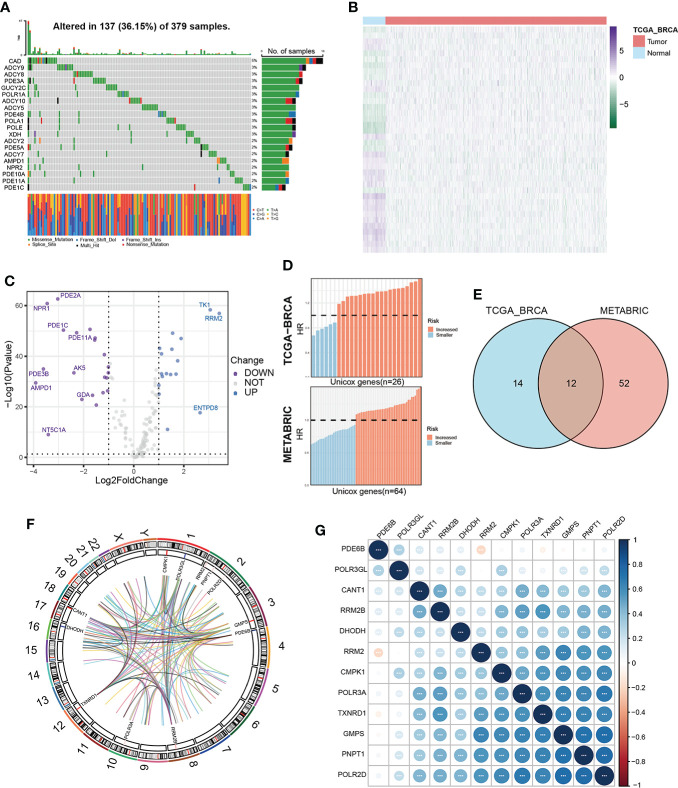
Characterization of prognosis-related PMGs in BC patients. **(A)** The somatic mutation frequency of PMGs in the TCGA-BRCA cohort. **(B, C)** Differentially expressed PMGs between normal and tumor tissues in TCGA-BRCA were shown in a heatmap and a volcano plot successively. **(D)** Prognostic PMGs were screened by the univariate Cox analysis in TCGA-BRCA and METABRIC severally. **(E)** A Venn diagram to obtain 12 overlapping prognostic PMGs. **(F)** A circos plot depicting the chromosomal location of the 12 prognostic PMGs and the chromosomal location of their interacting genes and describing the expression levels of the 12 PMGs. **(G)** The correlation characteristics between the 12 prognostic PMGs in TCGA-BRCA was revealed with a correlation matrix plot.

Our study flow chart is shown in **Figure 1**. Initially, we evaluated 163 global variations in PMGs in somatic mutations of the TCGA-BRCA cohort. The waterfall plot displayed the top 20 genes, which had the highest frequencies of somatic mutations **(** **Figure 2A** **)**. In addition, after comparing the mRNA expression levels of PMGs in the TCGA-BRCA cohort between the BC specimens and normal breast specimens using |log2FC|>1 and FDR<0.05 as thresholds, the results were attained, which are displayed herein by heatmap **(** **Figure 2B** **)** and a volcano plot **(** **Figure 2C** **)**. As shown in the volcano plot, the PMGs of which |log2FC|>2 were marked with the symbol names. In addition, to identify PMGs significantly associated with BC prognosis for subsequent model construction, we used the univariate Cox regression analysis to screen OS-associated genes in the TCGA-BRCA and METABRIC datasets **(** **Figure 2D** **)**, from which 26 and 64 important PMGs related to OS were obtained, respectively. After taking the intersection of the above results, we obtained 12 overlapping genes (CMPK1, POLR3GL, RRM2, PNPT1, POLR2D, GMPS, PDE6B, RRM2B, POLR3A, TXNRD1, DHODH, and CANT1) for the follow-up study **(** **Figure 2E** **)**. In addition, the chromosomal locations and expression levels of 12 genes were demonstrated by circos plots **(** **Figure 2F** **)**.The string connects the chromosomal location where the gene with which the 12 overlapping genes has a protein interaction is located. Finally, the association characteristics between these genes were unveiled by correlation matrix plots **(** **Figure 2G** **)**.

### Construction of prognostic signature pyrimidine metabolism-related in BC patients

**Figure 3:**
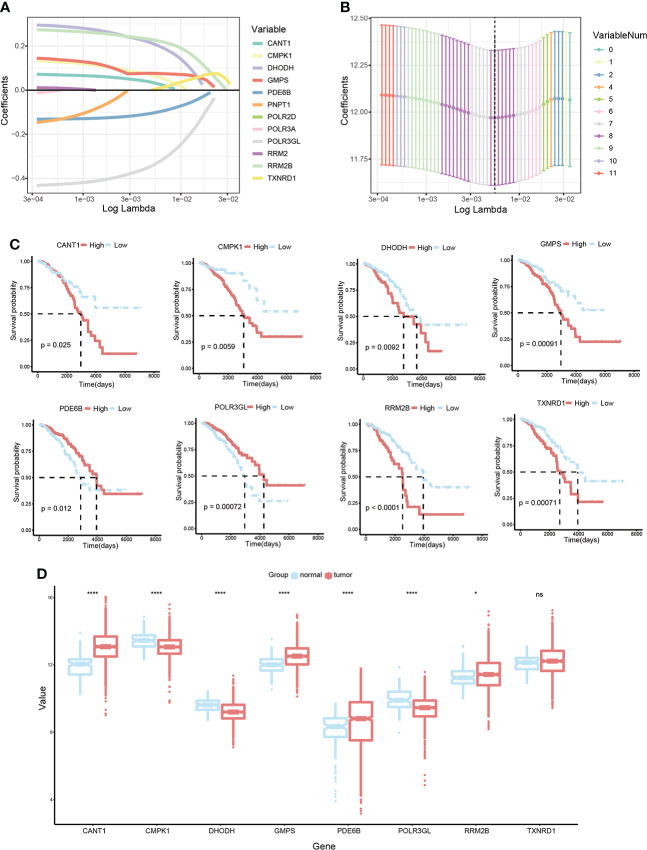
Development of a PMI signature in BC. **(A, B)** LASSO Cox regression analysis to select candidate PMGs for the signature. **(C)** The KM survival curves of eight selected PMGs based on expression levels and OS. **(D)** Estimation over the mRNA expression levels of eight signature-contained PMGs in the training cohort. *p< 0.05; **p< 0.01; ***p< 0.001; ****p< 0.0001; ns, no significance.

**Figure 4:**
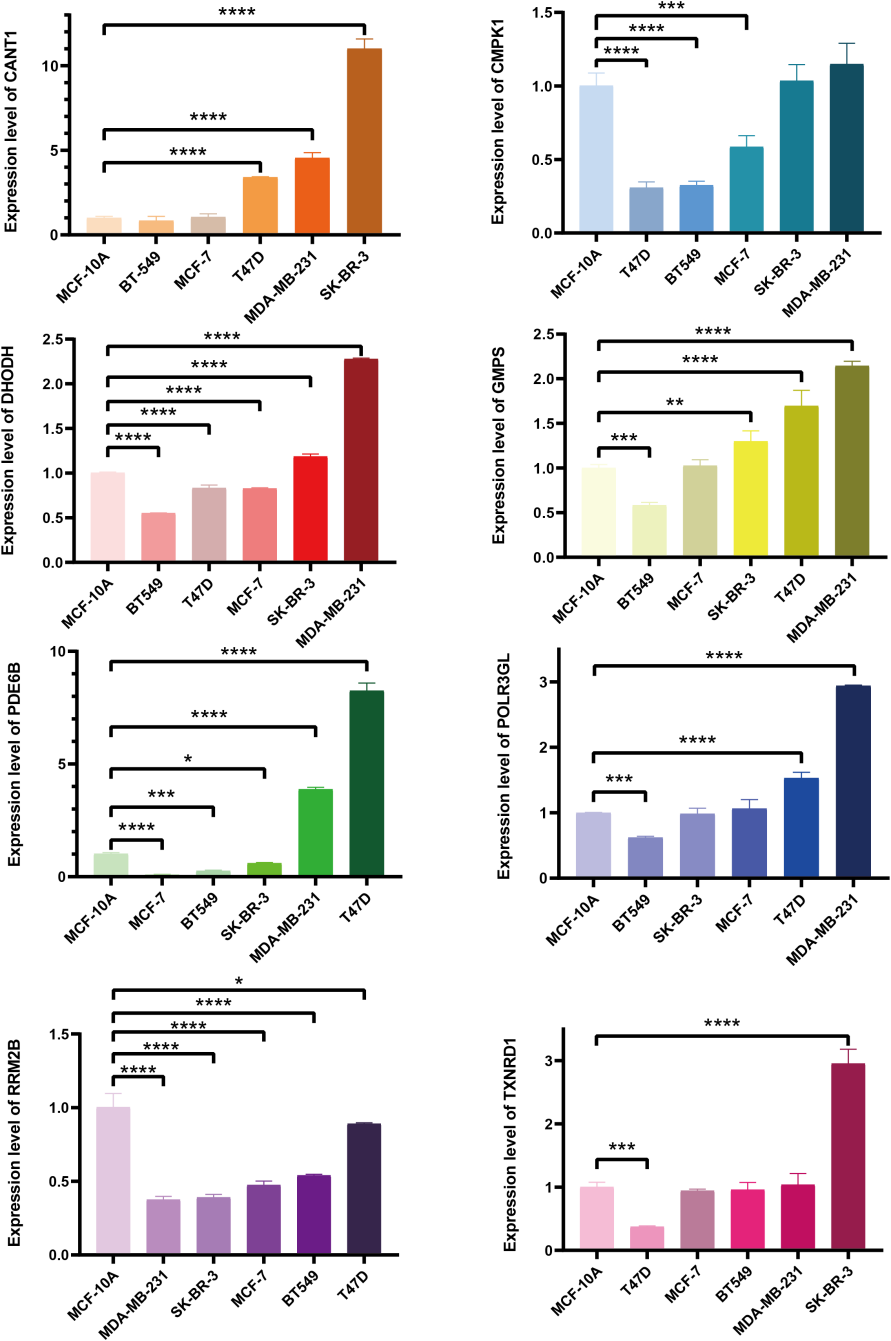
The mRNA expression levels of selected genes were detected in human BC cell lines and the normal mammary epithelial cell line. *p < 0.05; **p < 0.01; ***p < 0.001; ****p < 0.0001.

LASSO Cox regression analysis was conducted on 12 candidate genes in the TCGA-BRCA training dataset, and eight critical genes were identified to construct a prognosis signature, Pyrimidine Metabolic Index, designated as PMI **(** **Figures 3A, B** **)**, containing CANT1, CMPK1, DHODH, GMPS, PDE6B, POLR3GL, RRM2B, and TXNRD1. For further exploring the expression levels of each PMG and the ability to independently predict prognosis, and the relevance of PMGs expressions and OS was investigated using KM survival curves **(** **Figure 3C** **)**, the mRNA expression levels of BC versus normal tissues were demonstrated with boxplots **(** **Figure 3D** **)**. According to the results, the expressions of CANT1, GMPS, PDE6B, and RRM2B were notably elevated in BC, while the expressions of CMPK1, DHODH, and POLR3GL were significantly downregulated. In the KM analysis of OS, high expressions of CANT1, CMPK1, DHODH, GMPS, RRM2B, and TXNRD1, and downregulation of PDE6B and POLR3GL were markedly associated with a poor prognosis of BC, further confirming the reliability of selected PMGs. Furthermore, we detected the mRNA expression levels of signature-contained PMGs in common human BC cell lines, including MDA-MB-231, BT 549, SK-BR-3, MCF-7, and T47D **(** **Figure 4** **)**. The results indicated that the expression levels of CANT1, CMPK1, DHODH, GMPS, and PDE6B in the vast majority of human BC cell lines were consistent with the tissue expression levels in the database compared with mammary epithelial cell line MCF-10A. Ultimately, the prognostic signature – the PMI of each patient – was determined as follows:

PMI = Expression of CANT1 * 0.032209 + Expression of CMPK1 * 0.064783 + Expression of DHODH * 0.207055 + Expression of GMPS * 0.076095 - Expression of PDE6B * 0.10174 - Expression of POLR3GL * 0.33513 + Expression of RRM2B * 0.225794 + Expression of TXNRD1 * 0.004771

### Validation of signature based on eight pyrimidine metabolism-relevant genes

**Figure 5:**
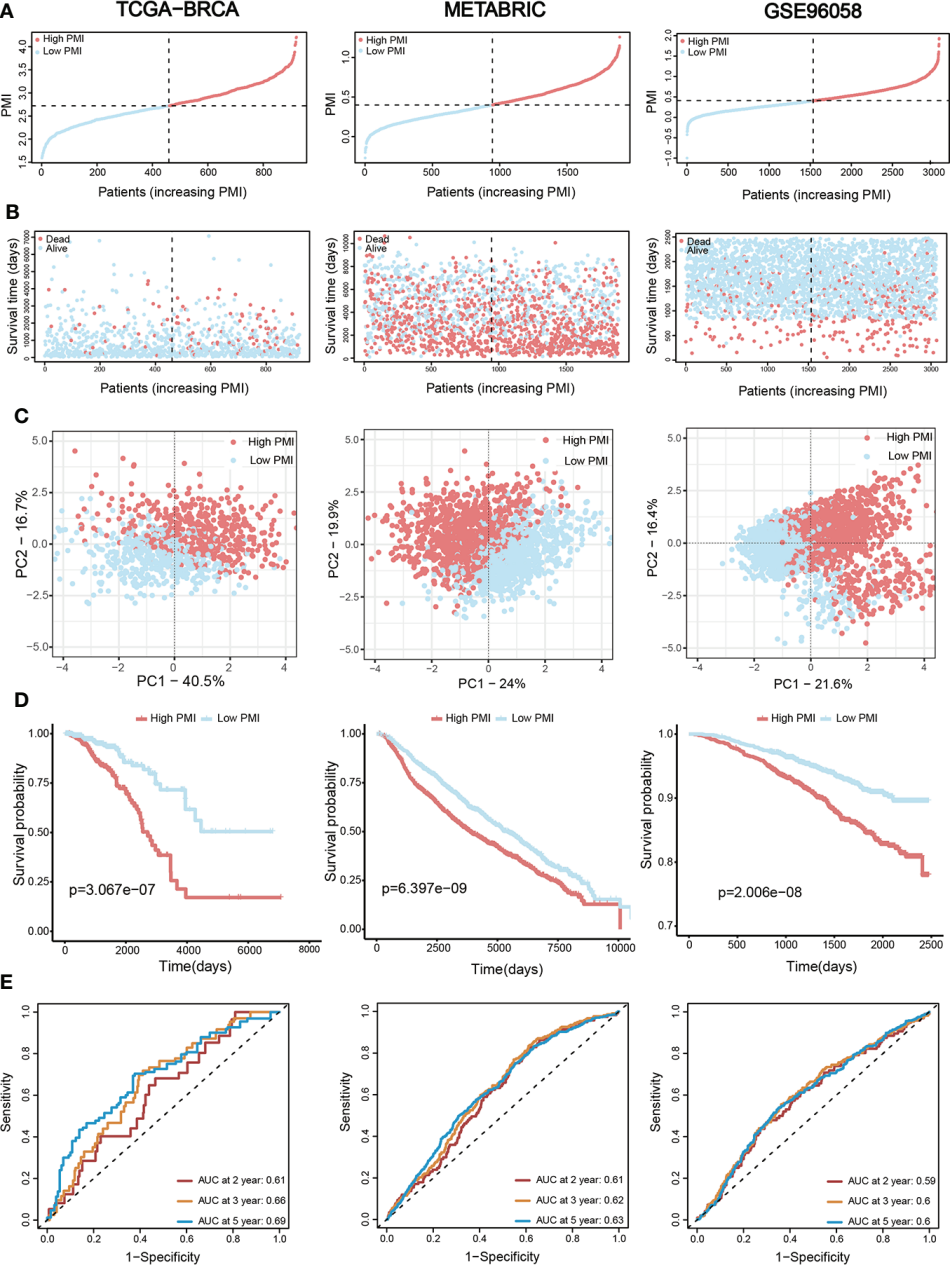
Assessment and verification of the efficiency of the PMI signature. **(A)** The increasing PMI score in TCGA-BRCA, METABRIC, and GSE96058. **(B)** Variations in the deaths of BC patients accompanied by PMI increasing. **(C)** The PCA analyses of high- and low-PMI clusters. **(D)** KM analyses of overall survival probabilities between the high- and low-PMI subgroups. **(E)** The ROC curves of the PMI-only model in predicting 2-, 3-, and 5-year OS of BC patients.

**Figure 6:**
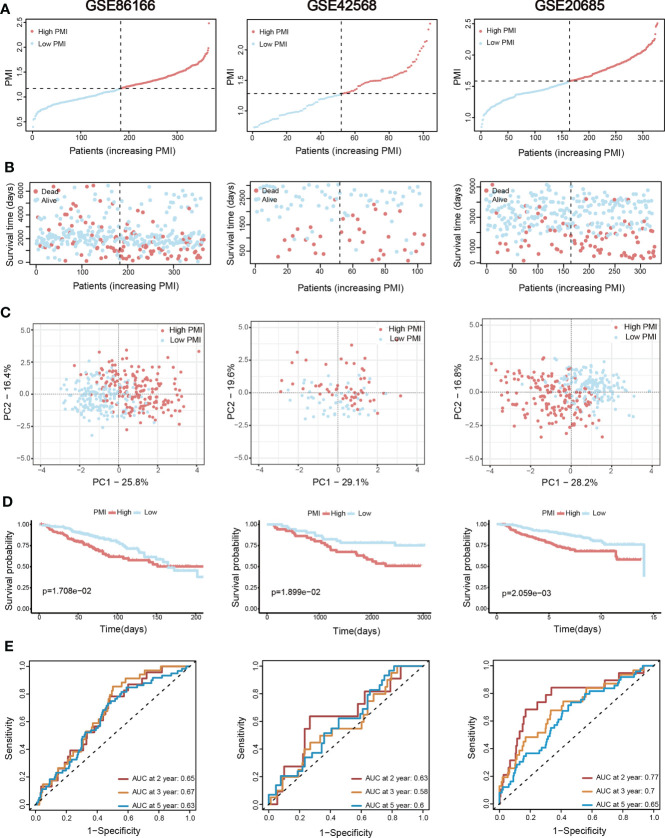
Additional three validation sets for the assessment and verification of the efficiency of the PMI signature. **(A)** The increasing PMI score in GSE86166, GSE42568, and GSE20685. **(B)** Variations in the deaths of BC patients accompanied by PMI increasing. **(C)** The PCA analyses of high- and low-PMI clusters. **(D)** KM analyses of overall survival probabilities between the high- and low-PMI subgroups. **(E)** The ROC curves of the PMI-only model in predicting 2-, 3-, and 5-year OS of BC patients.

BC patients from the TCGA-BRCA training set and two validation sets (METABRIC and GSE96058) were individually divided into high- and low-PMI subpopulations based on the median PMI value to further test the predictive accuracy of PMI in BC **(** **Figure 5A** **)**. As predicted, deaths of BC patients had risen with increasing PMI in all cohorts **(** **Figure 5B** **)**. Moreover, the distribution patterns of high- and low-PMI subgroups in a two-dimensional graph were visualized using PCA **(** **Figure 5C** **)**. Also, the KM survival analysis demonstrated that high-PMI patients tended to have a lower OS than patients with low PMI **(** **Figure 5D**, TCGA-BRCA, p = 3.067e−07; METABRIC, p = 6.397e−09; METABRIC, p< 0.0001; GSE96058, p = 2.006e^−08)^.Furthermore, the AUC curve confirmed that the PMI-only model was statistically significant for diagnosing the probability of survival in breast cancer patients **(** **Figure 5E** **)**.Finally, the predictive accuracy of PMI in BC was again validated in the GSE86166, GSE42568 and GSE20685 **(** **Figure 6** **)**.

### Comprehensive assessment of PMI and clinical parameters in BC patients

**Figure 7:**
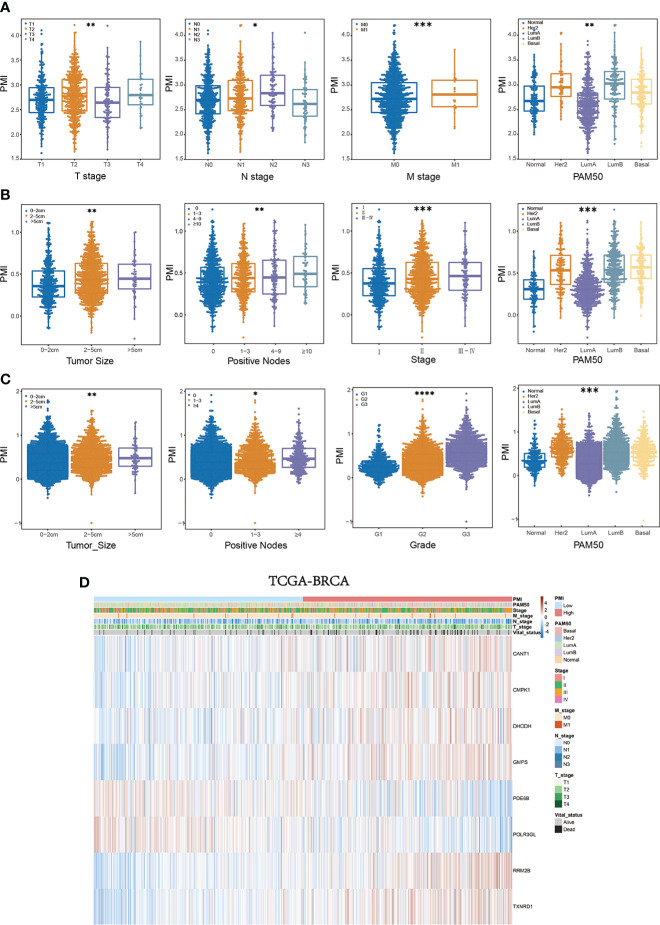
Systematic characterization of PMI and clinical variables among BC patients. **(A–C)** Beeswarm plots demonstrating the correlation between PMI levels and various degrees of diverse clinicopathological indicators in TCGA-BRCA **(A)**, METABRIC **(B)**, and GSE96058 **(C)** datasets. **(D)** Heatmaps incorporating PMI and clinical parameters in relation to gene expression levels in eight signature-included PMGs in TCGA-BRCA *p< 0.05; **p< 0.01; ***p< 0.001; ****p< 0.0001.

We further determined the relationship between PMI and clinical characteristics to elucidate the ability of PMI to predict other clinical factors. In the training set, significant variations existed in PMI at different levels of survival status, T stage, N stage, M stage, and PAM50 subtypes (all p< 0.05), which demonstrated that high PMI might be relevant to the severity of the clinical parameters described above **(** **Figure 7A** **)**. Similarly, remarkable discrepancies were reaffirmed between PMI and various levels for diverse clinical parameters, including unfavorable survival probability, larger tumor size, more positive nodes, severer stage, and PAM50 subtype in high-PMI patients of the METABRIC cohort **(** **Figure 7B** **)** and worse survival status, more massive tumor, more metastatic lymph nodes, higher tumor grade, and PAM50 subtype in high-PMI patients of the GSE96058 cohort **(** **Figure 7C** **)**. The integrated correlation analyses were also displayed with heatmaps in TCGA cohort **(** **Figure 7D** **)**, METABRIC cohort and GSE96058 cohort **(** **Supplementary Figure 2** **)**.

### Development and assessment of clinicopathological nomogram related to pyrimidine metabolism

**Figure 8:**
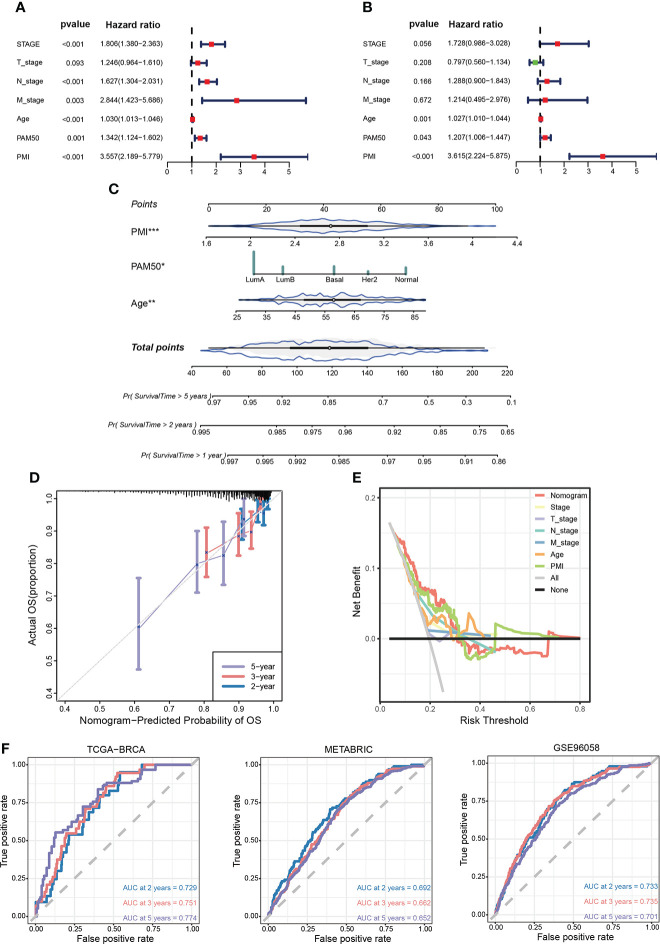
Establishment of a prognosis-related nomogram model based on PMI. **(A)** Univariate Cox regression analysis of PMI and clinicopathological characteristic. **(B)** Multivariate Cox regression analysis of PMI and clinicopathological characteristic. **(C)** Construction of a nomogram to predict OS of BC patients from TCGA-BRCA. **(D)** The calibration curve to estimate prediction accuracy of the nomogram based on the agreement of predicted OS with actual OS. **(E)** The decision curve to evaluate the clinical decision effectiveness of the nomogram against other separate clinical parameters. **(F)** The ROC curves and AUC values demonstrated favorable competence of the nomogram in predicting 2-, 3-, and 5-year OS of BC patients. *p < 0.05; **p < 0.01; ***p < 0.001.

To ascertain whether PMI signature could serve as an independent predictor for prognoses of BC patients, we conducted univariate and multivariate Cox regression analyses in the TCGA-BRCA cohort. The results demonstrated that the T stage, N stage, M stage, age, PAM50 subtype, and PMI were notably correlated with OS in univariate COX analysis **(** **Figure 8A** **)**, while only age, PAM50 subtype, and PMI remained independently prognostic indicators in multivariate Cox analysis **(** **Figure 8B** **)**. Next, we developed a clinicopathological nomogram incorporating PMI, age, and PAM50 subtype to predict individual OS at 1, 2, and 5 years based on the above results **(** **Figure 8C** **)**. To further validate the predictive power of the model, calibration plots are depicted to confirm the predictive consistency **(** **Figure 8D** **)**. Subsequently, to compare the discrepancy in the predictive power between nomogram and single independent clinical parameters, decision curves were drawn to demonstrate that nomogram yielded greater net benefits than single independent clinical features **(** **Figure 8E** **)**. To demonstrate the predictive power of the model more visually in multiple aspects, AUC analyses were conducted **(** **Figure 8F** **)**. The results showed that the PMI model had significantly predictive efficacy in patients of both training and validation cohorts.

### Gene Set Enrichment Analysis between two PMI groups

**Figure 9:**
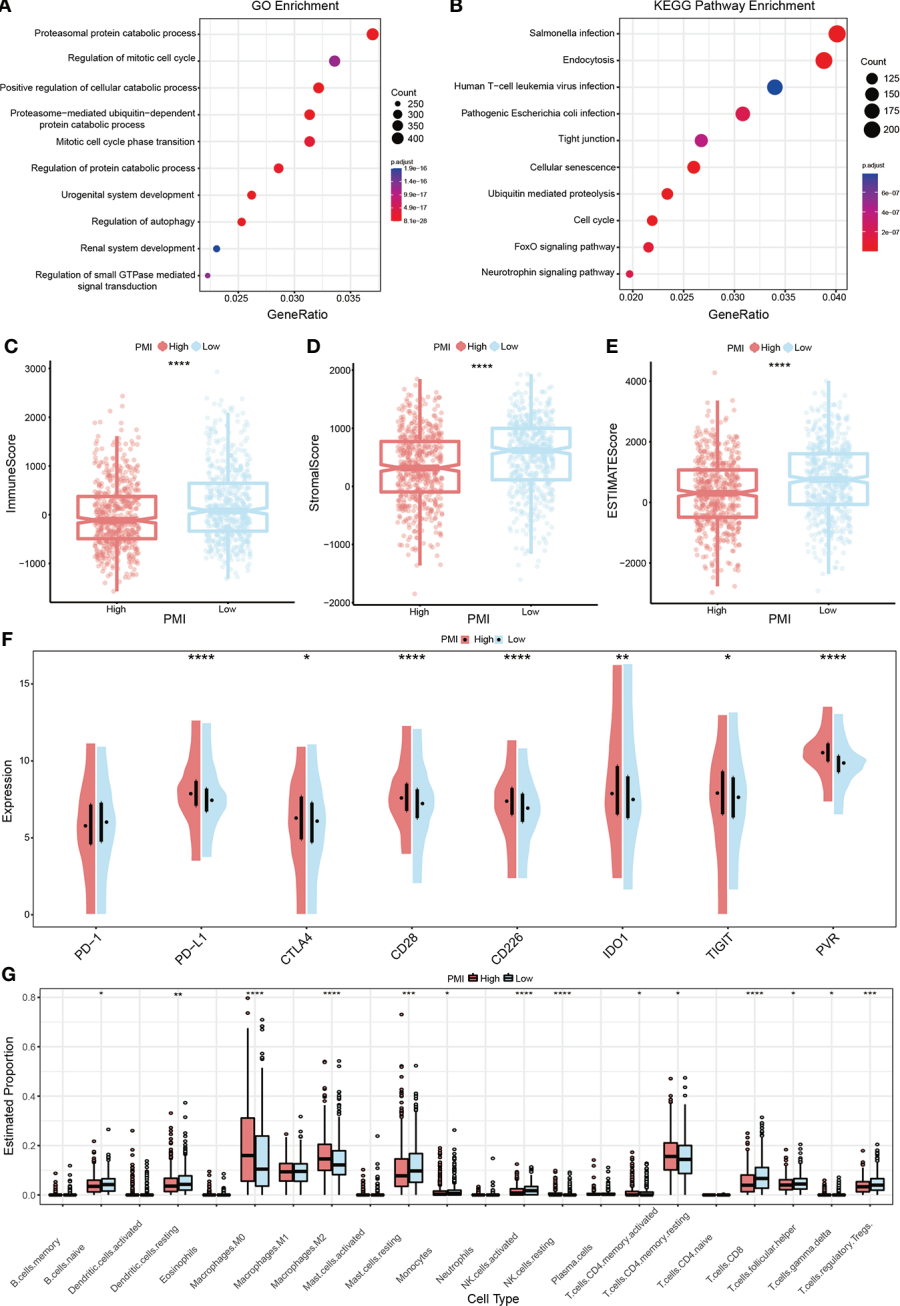
PMI-based GSEA enrichment analysis and TME assessment in BC. **(A, B)** Presentation of the top 10 differential pathways from GO **(A)** and KEGG **(B)** enrichment analysis results in TCGA-BRCA. **(C–E)** Variance of immune score **(C)**, stromal score **(D)**, and ESTIMATE score **(E)** in different PMI groups. **(F)** Differences in the mRNA expression levels of eight well-known immune checkpoints between different PMI groups. **(G)** Boxplots were used to depict the discrepancies in the infiltration extent of 22 immune cells between different PMI groups among BC patients. *p< 0.05; **p< 0.01; ***p< 0.001; ****p< 0.0001.

GSEA analysis in the training set was performed to identify whether the enriched signaling pathways and biological functions were distinguished between the high- and low-PMI groups. The results using the ontology gene sets indicated that proteasomal protein catabolic process, regulation of mitotic cell cycle, positive regulation of cellular catabolic process, proteasome-mediated ubiquitin-dependent protein catabolic process, and mitotic cell cycle phase transition were mainly enriched in the high-PMI group **(** **Figure 9A** **)**, when using KEGG gene sets salmonella infection, endocytosis, human T-cell leukemia virus 1 infection, pathogenic *Escherichia coli* infection and tight junction were enriched in the high-PMI group **(** **Figure 9B** **)**.

### Latent implications of PMI-based immunotherapy and TME landscape estimation

Tumor immune microenvironment landscape and characteristics are increasingly relevant to tumor development and subsequent therapeutic aspects. Therefore, the ESTIMATE algorithm was applied to evaluate and quantify TME by calculating the immune score, stromal score, and ESTIMATE score. The results demonstrated that the low PMI group obtained higher scores above shown in comparison to the high PMI group **(** **Figures 9C–E** **)**. In addition, as an up-and-coming target for immunotherapy in the 21st century, immune checkpoints are playing an essential role in clinical BC treatment protocols. Thus, a comparison of candidate immune checkpoint with mRNA expression levels in both PMI groups showed that PD-L1, CTLA4, CD28, CD226, IDO1, TIGIT, and PVR except PD-1 were dramatically increased in the high-PMI group **(** **Figure 9F** **)**, intimating that patients with a high PMI may achieve more enhanced responses to immunotherapy against the above checkpoints. The results were also very close in the other two validation groups **(** **Supplementary Figure 3** **)**.

It is unsurprising to conclude from the above results that TME differs significantly between different PMI groups. To further investigate the variation of immune cell infiltration in TME of BC in different PMI groups, the CIBERSORT algorithm was applied. The results demonstrated that macrophages M0 and M2, resting NK cells, activated memory CD4+ T cells, and resting memory CD4+ T cells were markedly elevated in the high-PMI group, while naive B cells, resting dendritic cells, resting mast cells, monocytes, activated NK cells, CD8+ T cells, follicular helper T cells, gamma delta T cells, and regulatory T cells were considerably intensified in the low-PMI group **(** **Figure 9G** **)**. Furthermore, we also conducted the identical analysis of the other two validation sets **(** **Supplementary Figure 4** **).** In both the training and the other two validation sets, macrophages M0 and activated memory CD4+ T cells were intensified significantly, while resting mast cells and gamma delta T cells were attenuated in the high-PMI group.

Altogether, the above results unveiled a significant correlation and complexity between TME and pyrimidine metabolism, which deserves further in-depth study.

**Figure 10:**
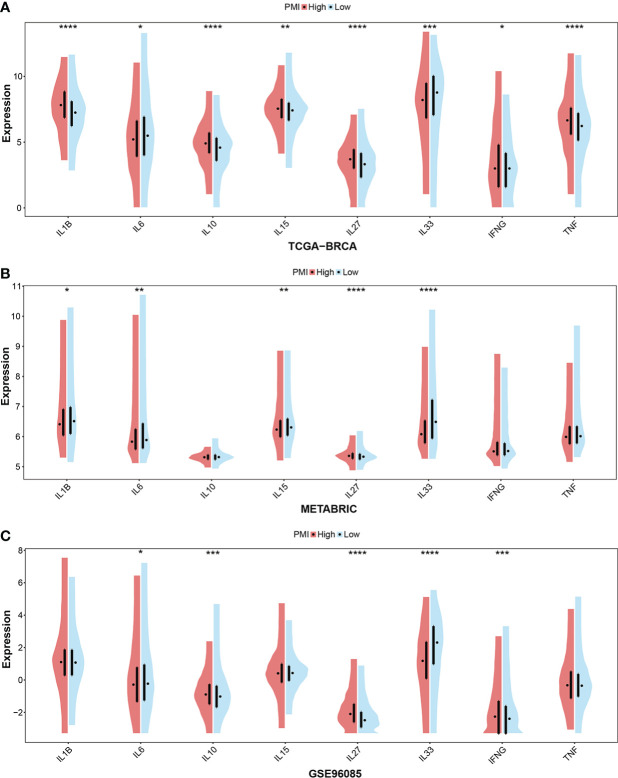
Investigation of essential cytokines in TME according to PMI levels. Comparison between the mRNA expression levels of IL-1B, IL-6, IL-10, IL-15, IL-27, IL-33, IFNG, and TNF between high- and low-PMI groups were explored in TCGA-BRCA **(A)**, METABRIC **(B)**, and GSE96058 **(C)** datasets separately. *p< 0.05; **p< 0.01; ***p< 0.001; ****p< 0.0001.

In addition, cytokines are a critical component of immune TME. Given this, we further scrutinized the correlation between PMI signature and cytokines, which included the essential cytokines of TME, IL-1B, IL-6, IL-10, IL-15, IL-27, IL-33, INFG, and TNF. In the TCGA-BRCA, METABRIC and GSE96058 cohorts selected, the boxplots demonstrated that the expressions of IL-27 and INFG were consistently elevated in the high-PMI group, those of IL-6 and IL-33 were consistently declined in the high-PMI group **(** **Figure 10** **)**. The results illustrated that PMI signature were significantly related to cytokines in the TME.

### Prognostic investigation of PMI in BC patients receiving different treatments

**Figure 11:**
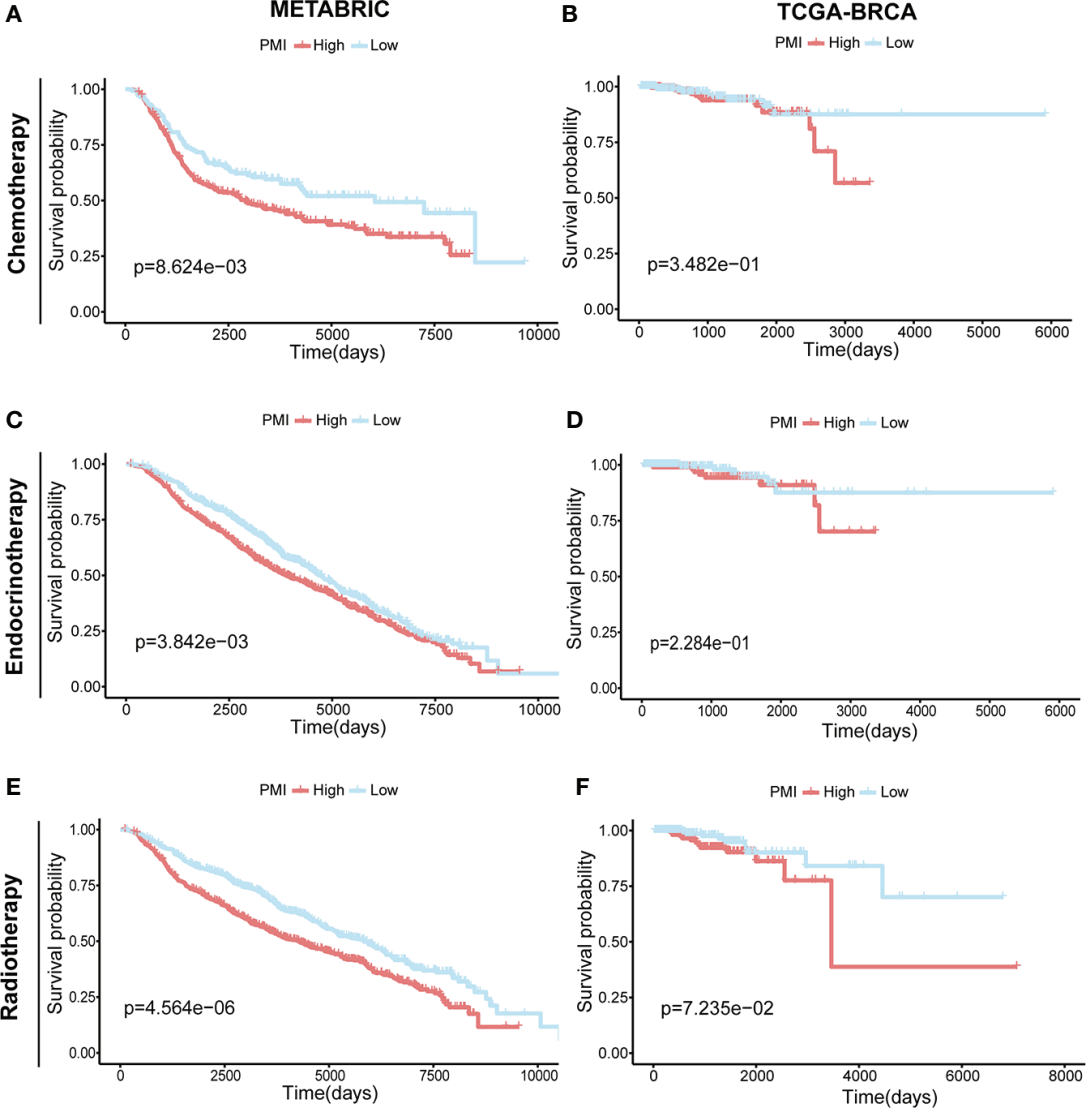
Prognostic Investigation of PMI in BC Patients Receiving Different Treatments.KM analyses of overall survival probabilities between the high- and low-PMI subgroups in BC patients undergoing chemotherapy **(A, B)**, endocrinotherapy **(C, D)** and radiotherapy **(E, F)** in the METABRIC and TCGA-BRCA cohort, respectively.

In addition, we investigated the correlation between pyrimidine metabolism and therapeutic approaches in BC patients. In the METABRIC cohort, the BC patients receiving chemotherapy (n = 396) **(** **Figure 11A** **)**, endocrinotherapy (n = 1168) **(** **Figure 11C** **)** or radiotherapy (n = 1134) **(** **Figure 11E** **)** both had poorer prognosis in high PMI group. While in TCGA-BRCA cohort, there was no statistically significant prognosis between the high- and low- PMI groups who were undergoing chemotherapy (n = 512) **(** **Figure 11B** **)**, endocrinotherapy (n = 470) **(** **Figure 11D** **)** or radiotherapy (n = 506) **(** **Figure 11F** **)**. We hypothesized that this result was due to a lack of adequate case samples or that patients with high PMI required chemotherapy, radiation and endocrine therapy to achieve a similar prognosis as patients with low PMI.

## Discussion

In recent decades, considerable feats have been achieved in the treatment and survival improvement of BC (31). Some BC subtypes have unique and efficacious treatment methods, for example, HER2-positive BC can be treated with trastuzumab-targeted therapy (32). Even so, a proportion of patients with BC still require more advanced therapy, and the emergence of pyrimidine metabolism brought a promising solution for this issue. As a complicated enzymatic network, pyrimidine metabolism incorporated nucleoside salvage, *de novo* nucleotide synthesis, and catalytic degradation of pyrimidines (33). Studies have demonstrated that a steady supply of dNTPs was fundamental to cancer cells, and consequently, the activation of PMGs has been regarded as indispensable for tumor growth (11). However, the prognostic implication of PMGs remains to be better elucidated in BC.

In this study, by applying the univariate COX regression analysis for these PMGs in both TCGA-BRCA and METABRIC datasets, 26 and 64 PMGs significantly associated with OS were obtained, respectively. Finally, 12 overlapping genes that were highly meaningful in predicting BC patients in the two cohorts were obtained. To maximize the validity of the following studies, we further performed the LASSO analysis of these 12 genes, and finally, eight optimal DEGs were chosen to establish a prognostic risk signature.

DHODH, a key enzyme in the *de novo* biosynthesis of pyrimidine nucleotides, has appeared as a therapeutic target for a variety of tumor treatments (34). The modulation of DHODH activity in cancer focused on activating the biosynthesis of *de novo* pyrimidine biosynthesis through CAD complex (35). Previous results have suggested that DHODH suppression was correlated with decreased cell proliferation in most cancer cell lines, which was consistent with it being a poor prognostic factor in our result. Notably, the upregulation of DHODH mRNA expression in BC showed a paradox, which may result from the existence of different molecular subtypes of BC and needs further investigation.

TXNRD1, the cytosolic selenoprotein thioredoxin reductase 1 (TrxR1), served as a central regulator of the thioredoxin system and may be inhibited pharmacologically to achieve selective killing of cancer cells (36, 37). More importantly, in certain cancer types, including gastric cancer and non-small cell lung cancer, TrxR1 appeared to be secreted into the serum and may be available as a biomarker of disease severity and responsiveness to treatment (38, 39). Our result revealed that TXNRD1 was overexpressed in BC with no significance, which obviously related to unfavorable survival. Regrettably, except for DHODH and TXNRD1, the relevance of other candidate genes to human cancer has not been reported. From the results we obtained above, upregulations of CANT1, GMPS, PDE6B, and RRM2B and downregulations of CMPK1 and POLR3GL were observed in human BC tissues. From the survival analyses, we found that high expressions of CANT1, GMPS, RRM2B, and CMPK1 and low expressions of PDE6B and POLR3GL were significantly correlated with low survival probabilities in BC patients. Technically speaking, the role of each selected gene in cancer required more exploration.

Basing on the eight prognostic genes, we then generated a risk signature, which was named “pyrimidine metabolic index” or PMI. Patients from the datasets selected were divided into high- and low-PMI groups independently. Surprisingly, BC patients with a high PMI exhibited a greater mortality rate compared with patients with low PMI, which implied that PMI had a promising value for predicting OS. In addition, the relationship between PMI and clinicopathological factors also demonstrated a remarkably strong correlation. In brief, PMI was positively linked with larger tumor, more lymph node metastasis, and a severer stage. Then, to examine whether PMI could serve as an independent prognostic indicator, we successively performed univariate and multivariate Cox regression analyses and successfully constructed a prognostic nomogram containing age, PAM50 subtype, and PMI. As can be seen from the calibration plot, the predicted probability derived from the nomogram was compatible with the observed probability. Similarly, the nomogram enhanced clinical benefit in comparison with conventional factors based on decision curves. Additionally, the nomogram demonstrated excellent performance in terms of ROC curves in both training and validation cohorts. At this point in the study, the reliability of this nomogram has been definitively confirmed.

Cancer metabolism has been widely studied, and cancer cells have a well-characterized metabolic phenotype that can profoundly affect the TME (40). Despite the growing attention to pyrimidine metabolism, there are still no available details on its relevance to immunotherapy. According to the results, patients with a low PMI obtained higher stromal scores, immune scores, and ESTIMATE scores, indicating that they might be more sensitive to immunotherapy. This would also explain the fact that patients with a high PMI have a worse prognosis since they could not benefit more from the immunotherapeutic treatment. Given that immune checkpoint inhibitors are essential in immunotherapy (41), we assessed the mRNA expression levels of eight selective immune checkpoints in BC patients, which revealed that the mRNA expression levels of all seven immune checkpoints, excluding PD-1 elevated statistically in the high-PMI group. In terms of this aspect, BC patients with a high PMI might have a stronger therapeutic response to drugs designed to inhibit immune checkpoints. Consequently, the estimation of immune infiltrating cells between the high- and low-PMI subgroups signified that macrophages M0 and M2, resting NK cells, activated memory CD4+ T cells, and resting memory CD4+ T cells were markedly activated in the high-PMI group, while naive B cells, resting dendritic cells, resting mast cells, monocytes, activated NK cells, CD8+ T cells, follicular helper T cells, gamma delta T cells, and regulatory T cells were notably strengthened in the low-PMI group. Higher infiltration levels of CD8+ T cells, and gamma delta T cells indicate better prognosis in BC patients (42, 43). This is consistent with our conclusion of poor prognosis in the high-PMI group. High infiltration of M2 cells is one of the risk factors for breast cancer (44), as seen in the TCGA-BRCA and METABRIC cohort, where distribution was significantly higher in the high-PMI group compared to the low-PMI group. The growth, differentiation, and activation of immune cells in TME were regulated by cytokines (45). Meanwhile, we investigated the expression levels of IL-1B, IL-6, IL-10, IL-15, IL-27, IL-33, INFG, and TNF between the high- and low-PMI groups. From the analysis results of training and two validation sets, elevated expressions of IL-27 and INFG and decreased expressions of IL-6 and IL-33 were stably presented in the high PMI group. Despite the primary demonstration of the relationship between pyrimidine metabolism and TME, it still requires further elucidation due to the functions of tumor-infiltrating immune cells are complicated (46, 47).

This study systematically analyzed pyrimidine metabolism-related transcriptomic profiling and created a prognostic signature PMI in BC patients. However, limitations still existed, and especially the profound mechanism of pyrimidine metabolism in TME demanded more exploration.

## Conclusion

Overall, our study identified a credible risk signature for BC patients based on PMGs. This signature was validated to have excellent predictive power and was identified as an independent prognostic factor for BC patients. We also dissected the unique relationship between this signature and immune TME. In conclusion, our study provides supportive implications for pyrimidine metabolism in BC research.

## Data availability statement

Publicly available datasets were analyzed in this study, the names of the repositories and accessions numbers are provided within the article/**Supplementary Materials**.

## Author contributions

All authors involved in the study has made a certain contribution, including designation (CZ, JX, and XD), data collection and analysis (YX and SY), manuscript drafting and revising (YL, WT, and XL) as well as study supervision (WD, RH, and WW). All authors have read and approved the final manuscript submitted.

## Funding

This study was supported by the Natural Science Foundation of Guangdong (No. 2020A1515010260, WW).

## Acknowledgments

We are very grateful for the data support provided by public databases and constructive and helpful comments from the reviewers.

## Conflict of interest

The authors declare that the research was conducted in the absence of any commercial or financial relationships that could be construed as a potential conflict of interest.

## Publisher’s note

All claims expressed in this article are solely those of the authors and do not necessarily represent those of their affiliated organizations, or those of the publisher, the editors and the reviewers. Any product that may be evaluated in this article, or claim that may be made by its manufacturer, is not guaranteed or endorsed by the publisher.

## Supplementary material

Supplementary Figure 1 The acquisition of 163 credible PMGs. **(A)** Venn diagram to acquire 163 overlapping PMGs in TCGA-BRCA, METABRIC and GSE96058 datasets. **(B)** The detailed information on 163 eligible PMGs.

Supplementary Figure 2 Heatmaps incorporating PMI and clinical parameters in relation to gene expression levels in eight signature-included PMGs in METABRIC **(A)** and GSE96058 **(B)**.

Supplementary Figure 3 The mRNA expression level of immune checkpoints in the METABRIC **(A)** and GSE96058 **(B)**.

Supplementary Figure 4 The TME landscapes between high- and low-PMI groups were estimated in the two validation sets. The boxplots were applied to display the infiltration context of 22 immune cells in METABRIC **(A)** and GSE96058 **(B)**.
