# Supplementary material for: Prognosis stratification in breast cancer and characterization of immunosuppressive microenvironment through a pyrimidine metabolism-related signature
Source: Front Immunol. 2022 Nov 29;13:1056680. doi: 10.3389/fimmu.2022.1056680 (PMC9745154; doi:10.3389/fimmu.2022.1056680)
Supplement: Supplementary file 1 [file Table_1.docx]

Supplementary Material

**SUPPLEMENTARY TABLE**

**Supplementary Table S1**. Primers sequences were shown.

| **Supplementary Table S1. Primers for qRT-PCR detection** | | |
| --- | --- | --- |
| CANT1 | Forward | GGAAGGTGATCCTGACGTTCT |
|  | Reverse | GTGTCATTGTACCAGTTGGCG |
| CMPK1 | Forward | GGAAGGCAGATGTATCTTTCGTT |
|  | Reverse | TGTTGACTGAAGGTAGGTCTGA |
| DHODH | Forward | GTTCTGGGCCATAAATTCCGA |
|  | Reverse | TCTGGGTCTAGGGTTTCCTTC |
| GMPS | Forward | ATGGCTCTGTGCAACGGAG |
|  | Reverse | CCTCACTCTTCGGTCTATGACT |
| PDE6B | Forward | GACGTGTGGTCTGTGCTGAT |
|  | Reverse | CTTGCCGTGGAGGATGTAGTC |
| POLR3GL | Forward | TTCCGCCCAGTACCTTTGC |
|  | Reverse | CTGGCCGGATGAAGTAGGG |
| RRM2B | Forward | ATTGGGCCTTGCGATGGATAG |
|  | Reverse | GAGTCCTGGCATAAGACCTCT |
|  |  |  |
| TXNRD1 | Forward | ATATGGCAAGAAGGTGATGGTCC |
|  | Reverse | GGGCTTGTCCTAACAAAGCTG |
| Β-actin | Forward | GAAATCGTGCGTGACATTAA |
|  | Reverse | AAGGAAGGCTGGAAGAGTG |
