# Supplementary material for: Prognosis stratification in breast cancer and characterization of immunosuppressive microenvironment through a pyrimidine metabolism-related signature
Source: Front Immunol. 2022 Nov 29;13:1056680. doi: 10.3389/fimmu.2022.1056680 (PMC9745154; doi:10.3389/fimmu.2022.1056680)
Supplement: Supplementary file 2 [file Table_2.docx]

Supplementary Material

**SUPPLEMENTARY FIGURE LEGEND**

**Supplementary Figure 1**. The acquisition of 163 credible PMGs. **(A)** Venn diagram to acquire 163 overlapping PMGs in TCGA-BRCA, METABRIC and GSE96058 datasets. **(B)** The detailed information on 163 eligible PMGs.

**Supplementary Figure 2**. Heatmaps incorporating PMI and clinical parameters in relation to gene expression levels in eight signature-included PMGs in METABRIC **(A)** and GSE96058 **(B)**.

**Supplementary Figure 3**. The mRNA expression level of immune checkpoints in the METABRIC **(A)** and GSE96058 **(B)**.

**Supplementary Figure 4**. The TME landscapes between high- and low-PMI groups were estimated in the two validation sets. The boxplots were applied to display the infiltration context of 22 immune cells in METABRIC **(A)** and GSE96058 **(B)**.
