# Supplementary figures and images for: Prognosis stratification in breast cancer and characterization of immunosuppressive microenvironment through a pyrimidine metabolism-related signature
Source: Front Immunol. 2022 Nov 29;13:1056680. doi: 10.3389/fimmu.2022.1056680 (PMC9745154; doi:10.3389/fimmu.2022.1056680)

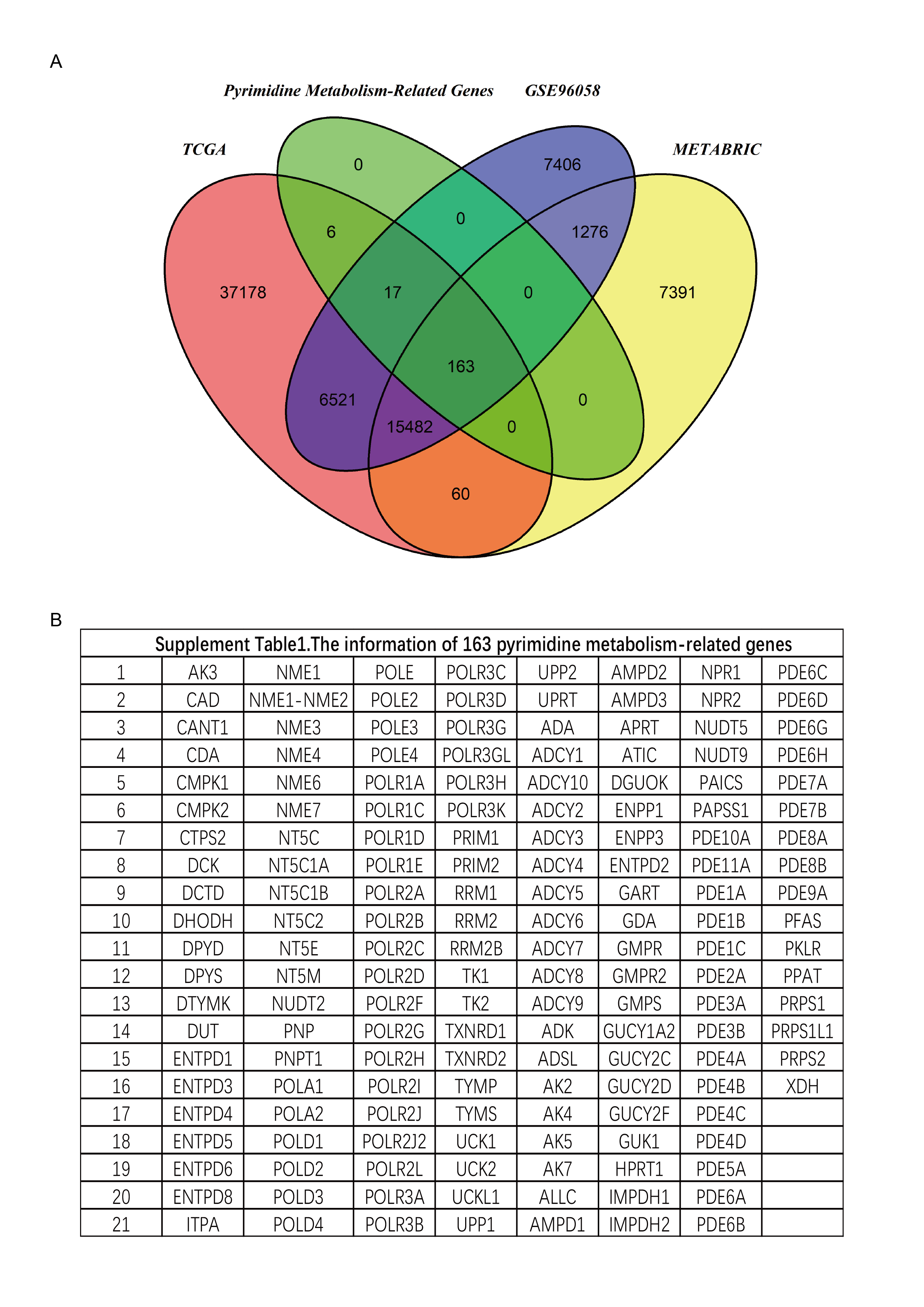

Supplement: Supplementary Figure 1 — The acquisition of 163 credible PMGs. (A) Venn diagram to acquire 163 overlapping PMGs in TCGA-BRCA, METABRIC and GSE96058 datasets. (B) The detailed information on 163 eligible PMGs. [file Image_1.tif]

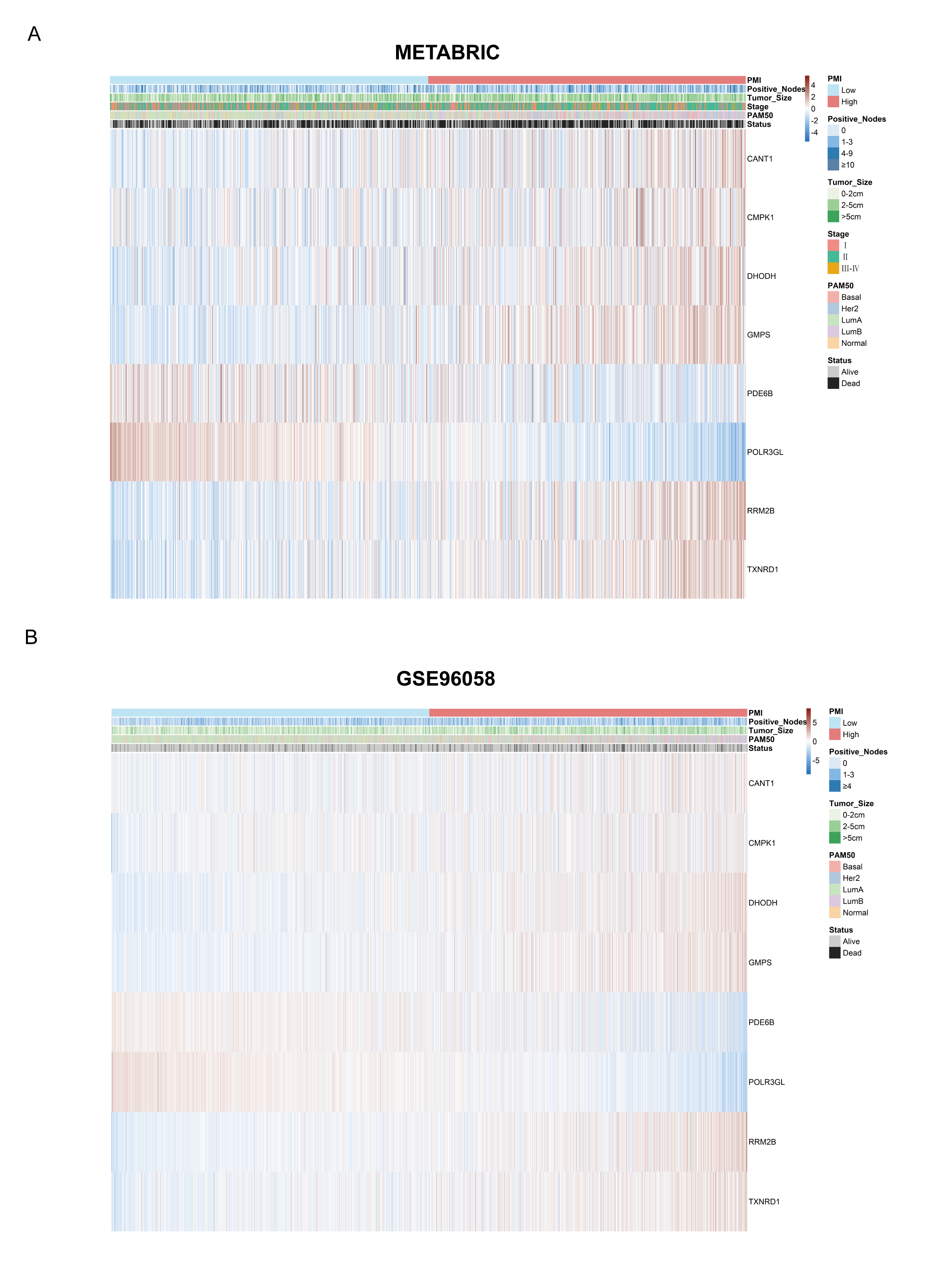

Supplement: Supplementary Figure 2 — Heatmaps incorporating PMI and clinical parameters in relation to gene expression levels in eight signature-included PMGs in METABRIC (A) and GSE96058 (B). [file Image_2.tif]

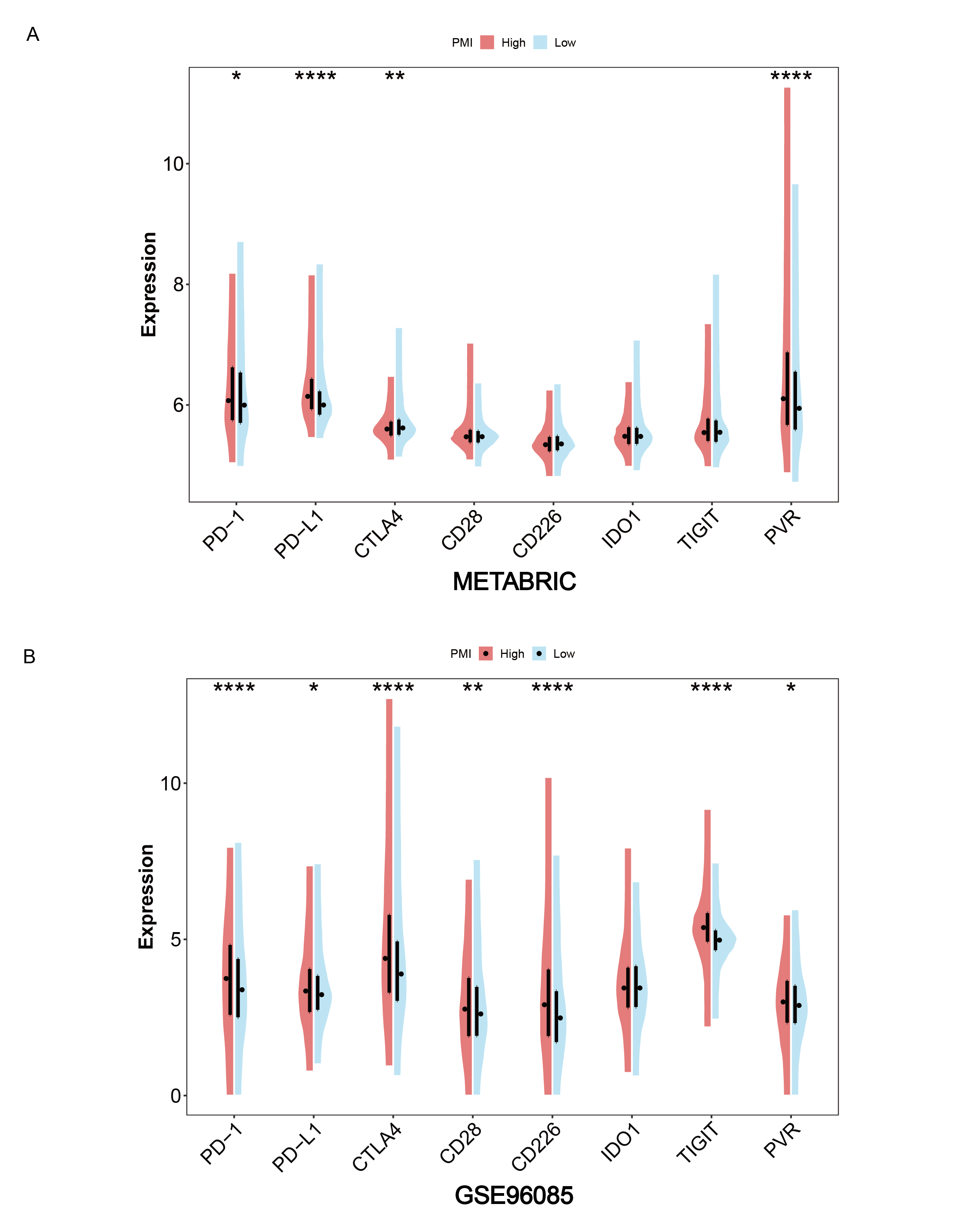

Supplement: Supplementary Figure 3 — The mRNA expression level of immune checkpoints in the METABRIC (A) and GSE96058 (B). [file Image_3.tif]

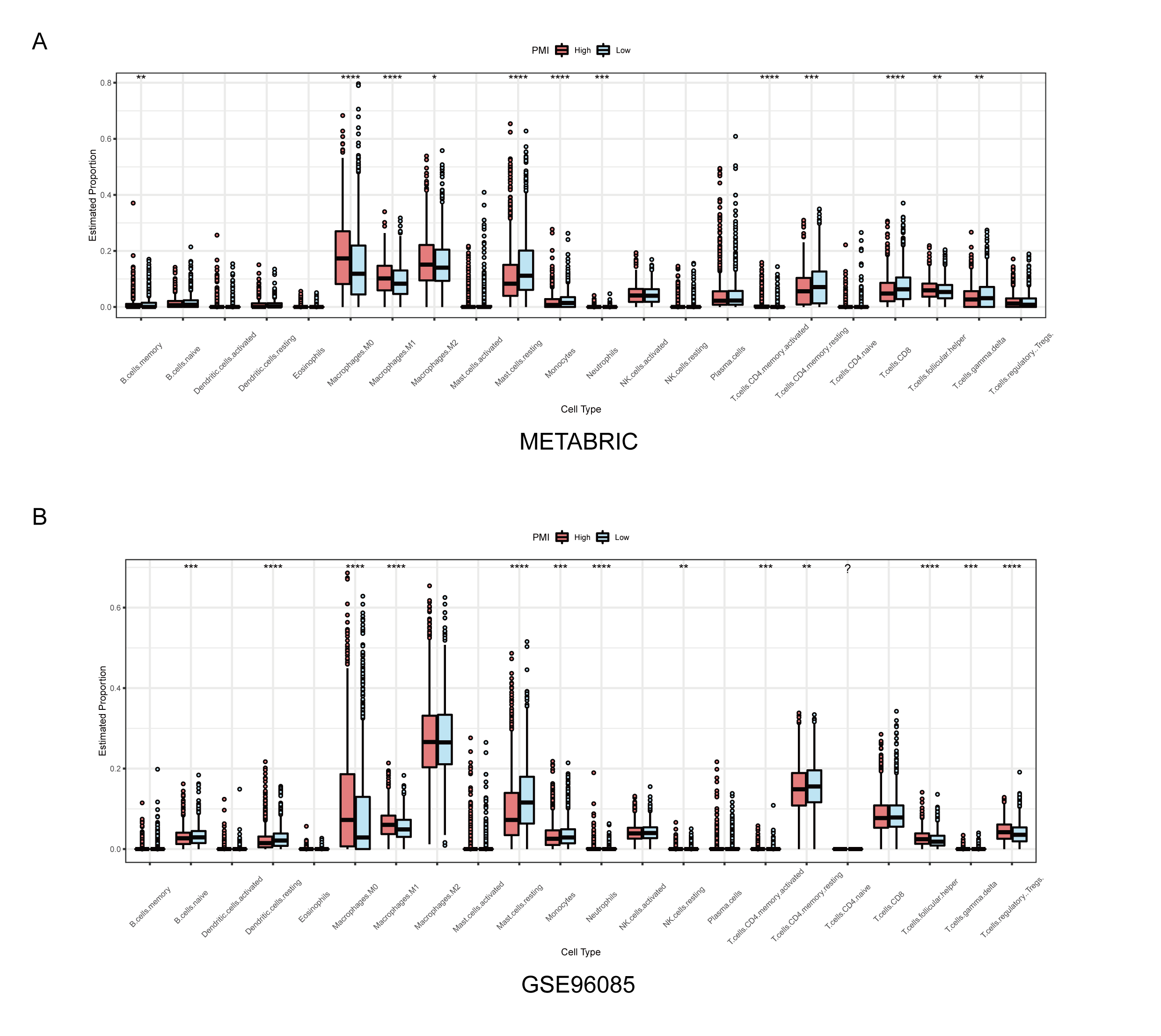

Supplement: Supplementary Figure 4 — The TME landscapes between high- and low-PMI groups were estimated in the two validation sets. The boxplots were applied to display the infiltration context of 22 immune cells in METABRIC (A) and GSE96058 (B). [file Image_4.tif]
